# Expectations on implementation of a health promotion practice using individually targeted lifestyle interventions in primary health care: a qualitative study

**DOI:** 10.1186/s12875-023-02079-5

**Published:** 2023-06-16

**Authors:** Emma Nilsing Strid, Lars Wallin, Ylva Nilsagård

**Affiliations:** 1 University Health Care Research Center, Faculty of Medicine and Health, Örebro University, Örebro, Sweden; 2 Department of Health and Welfare, Dalarna University, Falun, Sweden

**Keywords:** Qualitative Research, Primary Health Care, Health Personnel, Implementation Science, Healthy Lifestyle, Change Management, Clinical Practice Guidelines

## Abstract

**Background:**

There is moderate to strong evidence of the effectiveness of health-promotion interventions, but implementation in routine primary health care (PHC) has been slow. In the Act in Time project implementation support is provided for a health promotion practice using individually targeted lifestyle interventions in a PHC setting. Identifying health care professionals’ (HCPs’) perceptions of barriers and facilitators helps adapt implementation activities and achieve a more successful implementation. This study aimed, at a pre-implementation stage, to describe the expectations of managers, appointed internal facilitators (IFs) and HCPs on implementing a healthy lifestyle-promoting practice in PHC.

**Methods:**

In this qualitative study five focus group discussions with 27 HCPs and 16 individual interviews with managers and appointed IFs were conducted at five PHC centres in central Sweden. The PHC centres are participating in the Act in Time project, evaluating the process and outcomes of a multifaceted implementation strategy for a healthy lifestyle-promoting practice. A deductive qualitative content analysis based on the Consolidated Framework for Implementation Research (CFIR) was followed using inductive analysis.

**Results:**

Twelve constructs from four of five CFIR domains were derived: Innovation characteristics, Outer setting, Inner setting, and Characteristics of individuals. These domains are related to the expectations of HCPs to implement a healthy lifestyle-promoting practice, which includes facilitating factors and barriers. The inductive analysis showed that the HCPs perceived a need for a health-promotion approach to PHC. It serves the needs of the patients and the expectations of the HCPs, but lifestyle interventions must be co-produced with the patient. The HCPs expected that changing routine practice into a healthy lifestyle-promoting practice would be challenging, requiring sustainability, improved structures, cooperation in inter-professional teams, and a common purpose. A collective understanding of the purpose of changing practice was vital to successful implementation.

**Conclusions:**

The HCPs valued implementing a healthy lifestyle-promoting practice in a PHC setting. However, changing routine methods was challenging, implying that the implementation strategy should address obstacles and facilitating factors identified by the HCPs.

**Trial registration:**

This study is part of the Act in Time project, registered in ClinicalTrials.gov with the number NCT04799860. Registered 03 March 2021.

**Supplementary Information:**

The online version contains supplementary material available at 10.1186/s12875-023-02079-5.

## Background

Lifestyle-related diseases (e.g., cardiovascular diseases, type 2 diabetes, chronic respiratory diseases, and certain forms of cancer) are still the leading causes of prolonged disability and premature death [1]. Tobacco use, harmful consumption of alcohol, low physical activity, and poor nutrition are the major risk factors for these chronic diseases and play a central role in health status and quality of life [2, 3]. In addition, these health risk behaviours form a cluster, creating a synergy effect that increases the risk of disease [4]. Moderate to strong evidence exists for the effectiveness of health promotion interventions [5–8], and clinical practice guidelines for health promotion and prevention of these lifestyle-related diseases have been established [9–11]. Still, the implementation in routine primary health care (PHC) practice has been slow and uneven [12, 13]. Many reasons for the lack of implementation of such interventions have been described at the organisational, structural, and professional levels [14–16], implying significant challenges for health care professionals (HCPs) to move towards a proactive PHC [14–19]. The challenges include time restraints, lack of prioritisation of health-behaviour change, perceptions of the HCP role, negative attitudes, and absence of skills and knowledge [14, 16]. Keyworth et al. suggest that future healthy lifestyle-promotion interventions in PHC should address HCPs’ perceptions of patient needs, facilitate HCPs to deliver interventions in routine practice, and provide training across diverse professional groups [14]. The impact of better health-promoting practices in PHC can improve population health, consistent with WHO’s proposal to prioritise healthy living in PHC [20].

Implementing evidence-based interventions and guidelines often requires a change in clinical practice, where the context in which the change occurs plays an important role [18, 21, 22]. Changing clinical practice also requires a change of HCPs’ behaviour. Methods that identify and prioritise barriers, link intervention components to the obstacles, and engage end-users have been suggested to change the behaviour of HCPs [23]. Moreover, change in health care practices may be more successful if the HCPs can influence the change process, are well prepared, and value the change [24].

**Fig. 1:**
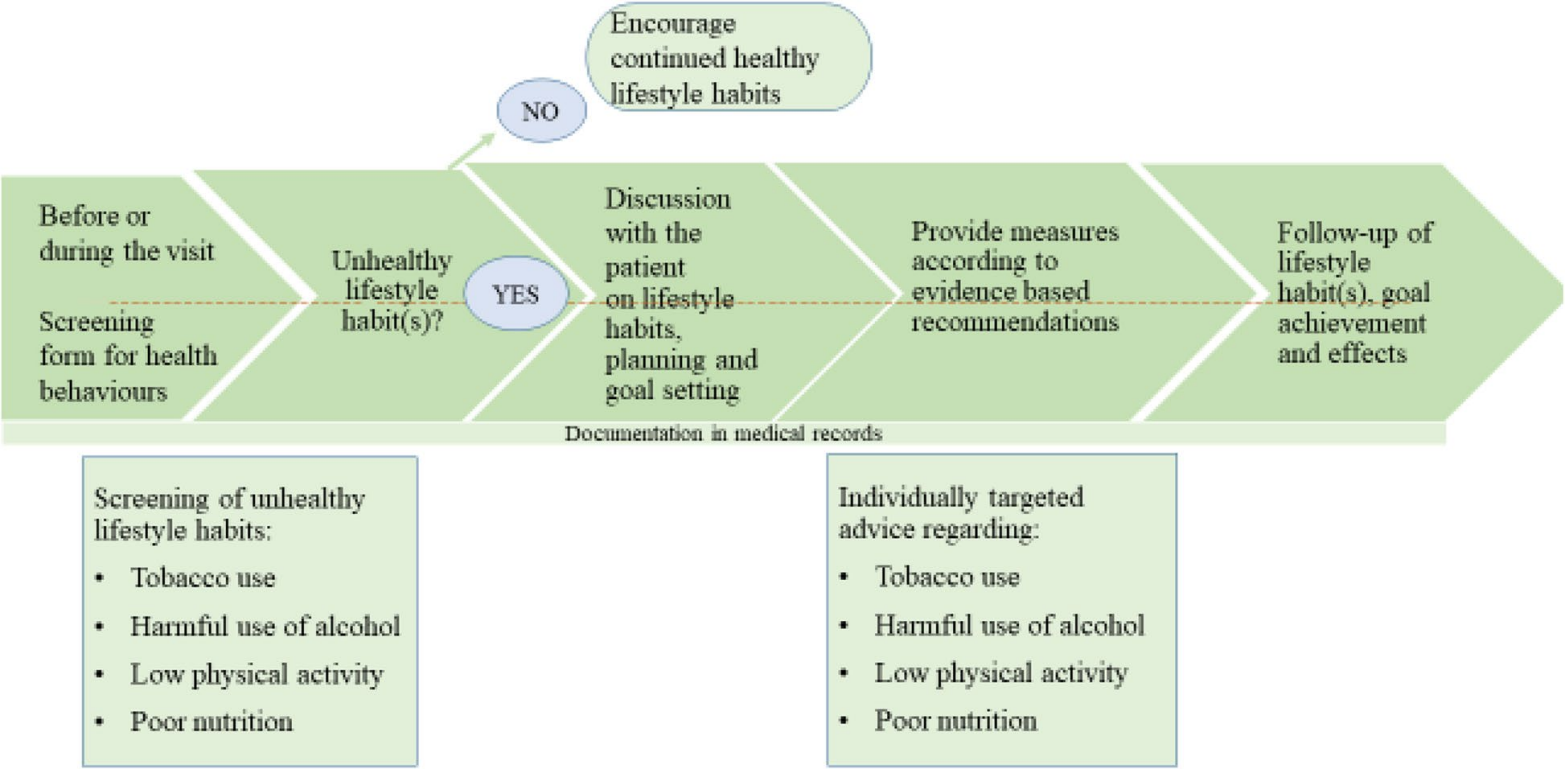
Overview of the clinical intervention, the health-promotion practice using individually targeted lifestyle interventions implemented in the Act in Time project [25]. The practice is based on Swedish national guidelines [11]

In the Act in Time project managers and HCPs in a Swedish PHC setting will be supported in implementing a healthy lifestyle-promoting practice [25]. Central components in the implementation strategy are external and internal facilitators (IFs), as described in the i-PARIHS framework [26, 27] and steps in the change leadership model [28, 29]. The practice is based on the Swedish national guideline for health promotion and disease prevention, targeting unhealthy lifestyle habits [11]. In this health-promoting practice, illustrated as a process in Fig. 1, HCPs will be expected to encourage patients to fill in a screening form regarding pre-visit health behaviours, discuss and offer individually targeted lifestyle advice to patients with unhealthy lifestyle habits, provide follow-ups, and document using the Swedish classification of health intervention codes in the medical record [11]. The classification codes are divided into qualified advice, advice and simple advice for lifestyle habits. Simple advice refers to information and short standardised advice on lifestyle habits. Advice and qualified advice is based on a person-centred dialogue with the patient. Qualified advice is more comprehensive, includes longer sessions, and requires professional competence in healthy lifestyle habits and skills in counselling and motivational interviewing [11]. Similar behaviour change principles have been described as the 5As (assess, advise, agree, assist, arrange), referring to effective activities supporting patients to change health-related behaviours [30].

One theoretical approach used in implementation science is determinant frameworks, describing general types of determinant that have been found (or hypothesised) to influence implementation processes and outcomes [31]. One of the most frequently used determinant frameworks is the Consolidated Framework for Implementation Research (CFIR) [22, 32–34]. The CFIR is a comprehensive, organising taxonomy of operationally defined constructs that may affect the implementation success of complex programmes [22]. The CFIR lists 39 key determinants of implementation organised into five domains based on context: Intervention characteristics, Outer setting, Inner setting, Characteristics of the individuals, and Implementation process. Each domain contains several constructs [22]. Some studies have described determinants for health promotion interventions [14–16, 35], but only a few have used a theoretical framework [17, 36, 37]. These studies used the CFIR after implementation to investigate what factors had influenced the implementation process. Collecting data on the target group’s expectations of clinical intervention and its perceptions of determinants for the implementation at a pre-implementation stage may enable the selection and adaption of implementation activities, which strengthens the opportunities to achieve more successful implementation [22, 31]. Using theoretical frameworks in empirical research may contribute to building an integrated body of knowledge on effective implementation [31]. Therefore, the CFIR was chosen as a theoretical framework in this study to explore determinants for implementing a healthy lifestyle-promoting practice before the implementation had begun. Collecting the expectations of managers and HCPs and their perceptions of barriers and facilitating factors regarding the implementation provides a base for the implementation strategy and an opportunity to select and adapt the implementation activities [31, 38, 39], and thus speed up implementing healthy lifestyle-promoting interventions in PHC.

### Aim

This study aims to describe the expectations of managers, appointed IFs, and HCPs on implementing a healthy lifestyle-promoting practice using individually targeted lifestyle interventions. The study was conducted before the implementation was initiated.

## Methods

### Design

The research used a qualitative descriptive design based on data collected through focus group discussions (FGDs) [40] and individual interviews [41]. Data were analysed using qualitative content analysis [42].

### Setting and recruitment of participants

This study was conducted pre-implementation as part of the Act in Time project, aiming to evaluate the process and outcomes of a multifaceted strategy for implementing a health-promoting practice using individually targeted lifestyle interventions in a PHC setting in central Sweden [25]. Five centres were voluntarily enrolled as intervention centres, representing a variation in size, socio-economic status, geographic location, and rural/urban division. A purposeful sample procedure was applied for interviews and FGDs with HCPs affiliated with these centres [43]. We sent written information about the study at each intervention centre to the PHC managers and the HCPs selected as IFs. We approached six managers (five PHC managers and one unit manager) and 10 IFs for individual interviews. All agreed to participate. Fifteen of these participants were women. HCPs (general practitioners, physiotherapists, psychologists, social workers, district nurses, registered nurses, and assistant nurses) having patient visits were invited to take part in the FGDs. The invitation, including information about the study, was forwarded by the PHC managers or one of the IFs. One FGD was held at each centre. We sought to have a variety of professionals as participants but coming from the same centre in each group. Five FGDs were conducted with 26 HCPs (4–7 in each group). Characteristics of the participating HCPs are presented in Tables 1 and 2.

**Table 1:** Overview of participants in individual interviews

Informant	Age (years)	Profession	Years of clinical experience	Years working within PHC
Managers				
PHC centre 1, M1	52	Physiotherapist	28	28
PHC centre 1, M2	43	Nurse, Midwife	18	5
PHC centre 2, M	40	Physiotherapist	14	12
PHC centre 3, M	55	Care administrator	38	18
PHC centre 4, M	43	District nurse	19	13
PHC centre 5, M	54	District nurse	28	18
Internal facilitators				
IF 1	33	Midwife	4	2,5
IF 2	36	District nurse	9	2
IF 3	31	Physiotherapist	9	8
IF 4	57	Nurse	21	1,5
IF 5	58	Nurse	32	15
IF 6	49	Physiotherapist	22	13
IF 7	58	District nurse	36	33
IF 8	47	Occupational therapist	25	1,5
IF 9	40	Physiotherapist	15	12
IF 10	30	District nurse	8	5

*Abbreviations*: *M* managers, *IF* internal facilitator, *PHC* primary health care. Two HCPs appointed as an IF at each PHC centre

**Table 2:** Overview of participants in focus group discussions (FGDs)

FGD	Total N	Age in years, min–max (mean)	Sex, M/W	Profession	Years of clinical experience, min–max	Years working within PHC, min–max
A	7	30–59 (48,4)	2/5	1 Midwife, 2 District nurses, 1 Physiotherapist, 2 General practitioners, 1 Counsellor	8–34	6 months -27
B	4	34–50 (41,5)	0/4	1 Physiotherapist, 1 Assistant nurse, 1 Midwife, 1 Counsellor	5-12^a^	4–7
C	4	49–63 (58,2)	0/4	1 Counsellor, 1 Nurse, 2 District nurses	11–42	2–22
D	6	25–65 (50,3)	1/5	1 Physiotherapist, 1 General practitioner, 1 Counsellor, 1 District nurse, 1 Assistant nurse, 1 Care administrator	5–38	3–36
E	5	26–49 (42,2)	2/3	1 Counsellor, 1 Physiotherapist, 1 District nurse, 2 General practitioners	3–12	3–9

*Abbreviations*: Sex *M* man, *W* woman, *PHC* primary health care

^a^missing information on one participant. One FGD was held at each PHC centre

### Data collection

We developed three semi-structured interview guides based on CFIR constructs as outlined in the CFIR interview guide tool (https://cfirguide.org) for individual interviews with managers, appointed IFs and FGDs with HCPs. The interview guides, slightly modified because of the explored perspective, targeted the participants’ expectations of the health-promoting practice concerning their working context and patients’ needs and their perceptions of the implementation and need for support (Additional file 1). The present article reports the findings on expectations and perceptions of implementing health-promoting practices. The questions were open-ended. Participants were encouraged to speak openly and share their perceptions and experiences. Concurrent probing questions were posed to deepen the discussions: “Could you elaborate on that?” The interview guides were pilot-tested with three PHC professionals not taking part in the project. Minor revisions, primarily regarding wording, were made. Six managers and 10 HCPs appointed as IFs were individually interviewed.

Data were collected from April 2021 to February 2022 by one author (ENS). Another author (YN) took part as an observer in the FGDs, taking field notes, observing the interaction and discussion flow, and posing complementary or clarifying questions. Because of the restrictions during the COVID-19 pandemic, most individual interviews were conducted by phone or digitally (Visiba Care). All FGDs were held at the PHC centres. FGDs were between 53 and 69 min and individual interviews lasted 35 to 67 min. The interviews and FGDs were digitally recorded and transcribed verbatim by two of the authors (ENS and YN) and a professional transcriber. To manage and code the data the transcribed texts were imported into NVivo 12 (QSR International, Melbourne, Australia).

### Data analysis

Data were analysed using deductive-inductive qualitative content analysis [42] guided by the CFIR [22]. Data analysis began after completing the interviews and FGDs at each intervention centre. Thus, data collection and analysis occurred parallel; however, no changes were made to the interview guides or the procedures. Two authors responsible for the analysis (ENS and YN) first read all transcripts to gain a sense of the whole. Next, a structured categorisation matrix was developed based on the domains and constructs in the CFIR to code the data [41, 42]. The coding matrix included definitions of the CFIR constructs, adapted to fit the study context. ENS and YN initially coded two transcripts jointly according to the CFIR constructs. The accuracy of the construct coding was then discussed with the third author (LW). After that, ENS and YN analysed three additional transcripts together to reach a mutual understanding of the data, CFIR constructs, and the coding strategy. ENS then coded the remaining transcripts.

Consensus discussions among all authors were held twice (when half of the data were coded and after all data were coded) to ensure that the meaning units were coded into the most appropriate CFIR constructs. Thereafter, the data in each construct were analysed using the principles of inductive content analysis [42], identifying sub-categories and categories. The inductive analysis was used to describe the expectations and perceptions of HCPs and managers regarding implementing the health-promotion practice of the CFIR constructs in greater depth. Codes from HCPs, managers, and IFs were analysed separately. Finally, all authors discussed the categorisation steps and agreed upon the final version of content and labels of categories and sub-categories. The data analysis process is described in Table 3. Quotations capturing the essence of the data were used to illustrate the categories [44]. These quotes were translated into English and then re-translated into Swedish to ensure consistency.

**Table 3:** Examples of the deductive analysis process include meaning units/quotations coded into the CFIR domain Outer setting and the construct Patient needs and resources, and then the inductive development of sub-categories and categories

**Meaning unit/quotation**	**Sub-category**	**Category**
We conduct this because we can see that people are feeling worse, their health is not good, more people are becoming sick and children are gaining weight. I can’t believe it. Already after one year, we had three patients with escalating weight curves. So we’ve got to start early with it.	A healthy lifestyle-promoting practice serves the needs of patients (facilitator)	A healthy lifestyle-promoting practice meets patients’ needs and should be co-produced with the patient
I think our patients will favour it because you expect that we in healthcare should talk [about lifestyle habits] (…). There's a lot of talk about it in society, lifestyle habits, health promotion, etc. So it will be natural that the primary care centre, which plays a big part, should also discuss it. So they [i.e. the patients] may wonder why we haven’t done this before.	Patients’ positive expectations of health promotion in PHC (facilitator)	
What the patient is comfortable with, not how the patient feels uncomfortable (…) Instead, evoke thoughts and maybe positively in the patient.	Patients’ needs and preferences must be considered while avoiding infringing on the patient’s autonomy (facilitator)	

### Ethical considerations

The Act in Time project was approved by the Swedish Ethical Review Authority (DNRs 2020–06956, 2021–00912 and 2021–05825-02). Participants in the interviews and FGDs received oral and written information about confidentiality, participant rights, and the project’s aim. All participants provided written informed consent, and the study complied with the ethical principles of the Helsinki Declaration [45]. The authors confirm all methods were conducted in accordance with relevant guidelines and regulations. The COREQ (COnsolidated criteria for REporting Qualitative research) checklist was used to ensure quality reporting [46].

## Results

The deductive analysis of the interviews and FGDs yielded 12 constructs from four CFIR domains (Innovation characteristics, Outer setting, Characteristics of individuals, and Inner setting) related to the HCPs’ expectations of implementing a healthy lifestyle-promoting practice. An inductive analysis was then employed, resulting in a range of categories and sub-categories describing the expectations of the HCPs more thoroughly. Potential facilitating factors and barriers were identified. The CFIR domains and constructs and the inductively developed categories and sub-categories are summarised in Table 4.

**Table 4:** Overview of CFIR domains and constructs and the inductively developed categories and sub-categories describing the HCPs’ expectations of implementing a healthy lifestyle-promoting practice

**CFIR domain**	**CFIR construct**	**Category and sub-category**
Innovation characteristics	Complexity	Changing to a healthy lifestyle-promoting practice is challenging and requires persistence to achieve sustainability • Changing to a healthy lifestyle-promoting practice is difficult (barrier) • Behaviour change is challenging to address and achieve as an HCP (barrier)
Outer setting	Patient needs and resources	A healthy lifestyle-promoting practice meets patients’ needs and should be co-produced with the patient • A healthy lifestyle-promoting practice serves the needs of the patients (facilitator) • Patients’ positive expectations of health promotion in PHC (facilitator) • Patients’ needs and preferences must be considered while avoiding infringing on the patient’s autonomy (facilitator)
Characteristics of individuals	Knowledge and beliefs	Understanding the purpose of changing to a healthy lifestyle-promoting practice is crucial • Shared beliefs and knowledge on the impact of health promotion (facilitator) • Insufficient knowledge of guidelines and tools for health promotion (barrier)
	Self-efficacy	• Beliefs in capabilities but uncertainty in practicalities (facilitator/barrier)
	Individual stage of change	• Variations from enthusiasm to potential reluctance in the ability to change health-promoting practice (facilitator/barrier)
	Other personal attributes	• Desire to work with health promotion and make a positive contribution (facilitator)
Inner setting	Networks and communications	Need structures, inter-professional teams and a sense of common purpose for a healthy lifestyle-promoting practice • Other HCPs’ competence and health-promoting practices are unknown (barrier)
	Implementation climate	• The necessity that all HCPs participate and work mutually towards the same goal (facilitator/barrier)
	- Tension for change	• A health-promoting practice is crucial for future PHC (facilitator) • A health-promoting practice is better than standard practice (facilitator)
	- Compatibility	• A healthy lifestyle-promoting practice is compatible with current practice but needs improved structures (facilitator/barrier)
	- Goals and feedback	• Importance of goals and feedback (barrier)

### Innovation characteristics

#### Changing to a healthy lifestyle-promoting practice is challenging, requiring persistence to achieve sustainability

The HCPs valued health promotion, describing it as part of the future, but implementing it would pose a challenge. *Changing to a healthy lifestyle-promoting practice is difficult*, especially if sustainability is a priority. There was a mutual understanding that implementing the interventions would take time. One participant described the work change as a gradual process in which health promotion occurs more frequently in PHC practice.

*“That we will hope because that's the meaning. If I think so? If I’m frank, I don’t think it's that easy. And to make it to something sustainable that isn’t just this year- that's what you hope for, and that is why it is so difficult. But we will hope for it” (IF 6, PHC centre 3).*

Several statements were related to the complexity of behaviour change, meaning that changes in *behaviour are difficult for HCPs to address*. Sustaining a shift in lifestyle habits in patients requires great effort and determination. The positive aspect of using a screening form for health behaviours to prepare the patient and the HCP for a dialogue on lifestyle habits was highlighted. However, the form should not have too many questions or be too short. Complexity issues were raised regarding technical aspects, such as the screening form not being integrated into the medical journal and being time-consuming to document when a paper version of the questionnaire was used. The HCPs emphasised that every HCP can ask questions and send out a screening form: the hard part is to support patients in changing their lifestyle habits.

“*What was it that made you not do this? And how would you change to make it happen? It’s a lot because you want to give as much information as possible and say you could do this, but it doesn’t work out. They know it, but they don’t get it right, and getting them on board from wanting to change lifestyle to actually do it is quite a lot of work” (FGD E, PHC centre 5).*

### Outer setting

#### A healthy lifestyle-promoting practice should meet patient needs and be co-produced with the patient

The participants expected that *a healthy lifestyle-promoting practice would serve their patients' needs, improve their health, and prevent* chronic diseases. The most important patients to reach were those with non-communicable diseases, although other patient groups seeking PHC could also be contacted. During the discussions, the participants reported that the demand for health-promotion practices might be underestimated. The challenges of cultural differences in lifestyle, beliefs, and knowledge underscored the need to reach out to all patients. The HCPs thought patients would change unhealthy lifestyle habits through the practices at the PHC centre. In the same way, patients with a healthy lifestyle could be encouraged to continue.

The HCPs expected that most *patients would have positive expectations of health promotion* and expect conversations about lifestyle and health in PHC, as these issues are frequently discussed in society and the media. They thought that most patients wanted to receive questions about their lifestyle. Although they want to learn more about living healthier, they seldom raise this issue themselves.

The HCPs expected few patients to feel provoked or that HCPs interfered with their lifestyle; instead, they cared about their health. However, some challenges were raised in discussions on lifestyle with patients seeking health care for reasons not necessarily associated with lifestyle factors and how a proactive practice met the needs of patients feeling well and without disease. Patients may wonder, “Why are you bringing this up” and perceive it as encroaching on their self-determination.

*“But then there’s this dilemma, as you say: I seek health care because of my back. We receive – we don’t call in patients for a health-promoting dialogue. Instead, they call us and have back pain, a stomach ache, a fever or something else. They’re coming (to us) seeking something… and then it's about… well, how can you get it in, so to say” (FGD D, PHC centre 4).*

The HCPs described concern about burdening the patient with shame or guilt or taking over the patient’s accountability. They noted that health promotion should be included in dialogue with the patient and that there must be different options to meet and *consider patients’ needs and preferences and avoid infringing on their autonomy.*

### Characteristics of individuals

#### Understanding the purpose of changing to a healthy lifestyle-promoting practice is crucial

The HCPs stressed the importance of *shared beliefs and knowledge on the impact of health promotion.* To trigger their inner motivation for changing practice, HCPs must understand why they should implement a healthy lifestyle-promoting practice.

*“I would like to emphasise how important this is and what it can lead to in public health… in a broader perspective. With better public health, the pressure [on PHC] may decrease. And prevention. Many of these patients might not have needed to seek health care if they’d received preventive help. This [health promotion] can make it easier in the future. So motivating why it should be done is especially important” (IF2, PHC centre 1).*

The HCPs discussed their impact on their professional role in communication with patients. By addressing lifestyle habits, they show their patients that this is important and allow them to reflect on their way of living. Sometimes these discussions may contribute to future change success. They acknowledged health promotion as an important component in patient assessment to understand their needs and offer tailored support and care. However, the participants felt that much more could be done and wanted to increase health promotion integration into regular practice: “*I think it’s needed a lot but that we have a massive job in front of us, which we also feel is fun and we want to do this for our patients*” (Manager, PHC centre 3).

There was *insufficient knowledge of guidelines and tools for health promotion*. In fact, most informants were unaware of the Swedish national clinical practice guidelines for health promotion and disease prevention. A few informants described using the screening form for lifestyle habits and supplementary questions, which helped them better understand the patient’s problem and the possibility of offering support. Most informants had little knowledge; some said they hesitated to use the screening form and stressed that they did not know what to do with the answers.

The HCPs expressed *beliefs in their capabilities but uncertainty in practicalities*. Concerns were raised about technical and practical aspects. Their beliefs in their abilities varied from feeling uncertain and uncomfortable about discussing lifestyle factors with their patients to being confident that they would easily master them. They expected their confidence to increase through education and practice. The HCPs felt confident in their ability to implement a healthier lifestyle-promoting practice. In addition, their managers described them as skilled in methods of improvement work. Also, the HCPs could apply knowledge from previous experiences with lifestyle behaviours to other lifestyle behaviours.

*“And the awareness that a minor change can greatly impact a person’s life, which I think everyone [i.e. HCP] has within their [professional] field. So one can understand this is not that difficult because I can already do it. I shall only apply it in a new field. And that this is my responsibility because the whole body is my responsibility” (Manager, PHC centre 1).*

The HCPs reported *variations from enthusiasm to potential reluctance to change health-promoting practices*. All colleagues may not share positive attitudes at the PHC centres. They expected questions if the timing for implementation was right, as well as personal barriers towards change and difficulties in changing routine professional practice. This scepticism was thought to be more pronounced at the beginning of the intervention. However, it was also because HCPs were not familiar with a healthy lifestyle-promoting practice or not feeling sufficiently skilled. The HCPs expected their colleagues to be in different stages of change (some being early adopters, some falling behind, and the majority in between). The HCPs explained working within tight professional boundaries in PHC, but to accomplish this practice change, they expected more teamwork in which all HCPs were included as equal partners. They discussed the importance of prioritising health promotion, explaining its benefits, and enjoying the results. Confidence in being amendable to change and loyal to each other was also expressed.

*“The biggest change is maybe this part. It’s often quite divided. This is the physician’s task. This is the nurse’s task. This is the counsellor’s, the physiotherapist’s, etc., task. (…). Because this would include all of us, it also implies a change in a way where we are all equal. And that’s new, and everyone will not appreciate it. Some will push it over on someone else; this is not mine, it´s the counsellor’s, and so on” (IF 8, PHC centre 4).*

Statements related to the HCPs’ traits entailed descriptions of their inner motivation towards health promotion, acknowledged as a facilitating factor. They described a *desire to work with health promotion and contribute positively.* Health promotion was considered meaningful and fulfilling, contributing to good patient care. Some HCPs described special interests in health promotion, declaring that this was one reason they worked in PHC. Health promotion was acknowledged from a salutogenic perspective, i.e., working with health and prevention, not merely a disease. The managers stated many HCPs had hoped for a more proactive and health-promoting practice in PHC and that previous lifestyle-promoting efforts had been popular and appreciated by patients and HCPs. The participants described engagement and motivation in starting something new. Knowledge of the benefits of a health-promoting practice and how it facilitates their work motivated the HCPs to adopt working with lifestyle interventions. The rationale for changing to a healthy lifestyle-promoting practice should not be derived from external pressure but driven by their inner motivation, i.e., the managers and HCPs see it as an essential component to better health care.

*“Regardless of how we twist and turn this, the driving force must come from here [i.e. PHC]. People from the outside cannot come and push; that won’t work” (Manager, PHC centre 1).*

### Inner setting

#### Need structures, inter-professional teams, and a common purpose for a healthy lifestyle-promoting practice

The HCPs expressed little knowledge of *other HCPs’ competence and health-promoting practices*. Managers did not know how much their employees worked with health promotion. The HCPs were unaware of how other health professionals worked with health promotion or what they did. They described them as working independently or intra-professionally but seldom inter-professionally. There was no overall picture of the competence or organisation of health promotion at the PHC centres. The HCPs called for more information and knowledge of each other’s competence to improve the processes of supporting patients in changing unhealthy lifestyle habits.

*“What do we offer a person who wants to quit smoking? How is it organised here at the centre? Do we refer them to hospital? How much [and] which drugs should you prescribe when supporting smoking cessation? To what extent do we do that here at the centre? There’s a good deal more information and knowledge I need” (IF 4, PHC centre 2).*

All HCPs had a shared understanding of working *towards the same goal*. Health promotion should be a theme and part of their commission and daily practice, regardless of profession. The HCPs outlined their belief that being unified and connected to their colleagues enables a change from a routine clinical practice to a more health-promoting approach. Communicating the same message throughout the care process would strengthen patient safety and provide equal care for all patients.

*“We should do the same thing. It shouldn’t matter if the patient meets me who is super interested in this, or someone else, some new colleague. It should be the same, in a way” (IF 10, PHC centre 5).*

Managers and IFs emphasised they needed time for reflection, planning, and considering other HCPs’ perspectives on how this health-promoting practice will affect them. They expected to work together and learn from each other.

The HCPs discussed tension for change, denoting *a health-promotion practice as crucial for future PHC*. From the HCPs’ perspective (i.e., primarily managers and IFs), health promotion must have a more significant role in PHC. The concern that there would be insufficient HCPs to care for the patient population led them to believe they would not solve this issue without a proactive and health-promoting practice. They feel they must work on health-promoting because of the need to improve health care. The HCPs also emphasised the need to examine health promotion from a broader perspective and to look ahead and not only focus on what is currently in demand. They noted that the *healthy lifestyle-promoting practice is better than the current care approach,* given that the basic health promotion offered today is insufficient.

*“We know the population is growing, that it will be more elderly and the people will be increasingly sicker at the same time as we in the healthcare will have no possibility to hire personnel corresponding to the need. So, if we look ahead, we know there will be a giant health care need and we will not sort it out” (Manager, PHC centre 1).*

The *healthy lifestyle-promoting practice was compatible with current practice but needed improved structures*. The HCPs underscored that health promotion is not a novel approach, as it is well-developed in some areas within PHC (e.g., paediatric and maternity care, specialist nurses, and psychosocial teams). HCPs advise and support healthy lifestyle habits within their expertise (e.g., physiotherapists on physical activity and physicians on alcohol and tobacco): a healthy lifestyle-promoting practice aligned well with how they already worked. However, there was no routine for health promotion at the PHC centres, and the participants emphasised the need for improvement.

*“I see lifestyle habits as the common thread running through everyone from counsellors to physios. Those in child and maternity care work a lot with it, so it’s in line with what we already do. So, it will be nothing new under the sun. I think all professionals know what to do with the lifestyle habits, but we don’t have a structured practice today” (IF 9, PHC centre 5).*

The HCPs stressed the *importance of goals and feedback*. Health promotion was acknowledged as a commission for PHC, but the goals for health promotion were still unclear. They warranted a clarification of the commission, the goals, and how they should work with health promotion. Follow-up and feedback were stressed, as well as opportunities to follow changes over time and analyse what may have affected the results. Good examples of previous successful work were regarded as inspiring. The best feedback was to see the results of their healthy lifestyle-promoting practice.

## Discussion

In this study 12 constructs from four CFIR domains were linked to the expectations of managers, appointed IFs and HCPs on implementing a healthy lifestyle-promoting practice using individually targeted lifestyle interventions. Their perceptions included facilitators and barriers in implementing a health promotion intervention in a PHC setting. The inductive analysis shows that the HCPs perceived a demand for a healthy lifestyle-promoting practice in PHC. In addition, it serves the patient’s needs and the expectations of the HCPs, but lifestyle interventions should be co-produced with the patient. The HCPs expected that changing routine practice into healthy lifestyle-promoting practice would be difficult, requiring persistence and improved structures to achieve sustainability. A collective understanding of changing the practice was deemed necessary for successful implementation. In line with previous studies the HCPs in the current study welcomed and valued an evidence-based healthy lifestyle-promoting practice that serves patient needs [19, 35]. Knowledge and beliefs about the consequences of unhealthy lifestyle habits have been acknowledged as a facilitating factor for implementing and sustaining health-promoting changes [14, 36]. The main research project will implement a healthy lifestyle-promoting practice based on Swedish national guidelines [11] in a PHC setting [25]. HCPs are more enthusiastic about implementing interventions underpinned by scientific evidence, largely because of the breakthroughs in evidence-based medicine.

The Swedish healthcare system is moving towards more integrated care [47], aligning with the global strategy of people-centred and integrated health care services [20]. Integrated care will be achieved by focusing on more proactive, health-promoting, person-centred health care with a coherent care chain. The ongoing work to enable this transformation may have influenced the informants and, thus, their narratives. The HCPs stressed the importance of addressing lifestyle habits in dialogue with the patient and shared decision making. This finding is consistent with the suggestion that lifestyle counselling should be based on a partnership between the patient and the HCP [48]. Shared decision making has been defined in a summary of NICE guidance as “a collaborative process that involves a person and their healthcare professional working together to reach a joint decision about care” [49]. This shared-based decision making is a key component of person-centred care. The joint decision-making process includes activities before, during, and after a patient visit, stressing potential risks, benefits, and consequences. The screening form for lifestyle behaviour sent out before a patient visit allows the patients to think about their lifestyle choices and prepare for a dialogue with the HCP about their habits, health, and needs. The HCP can prepare for the visit by taking part in the completed screening form. Shared decision making was expressed as desirable and included the opportunity to discuss personalised information about risks, the benefits of changing to healthier lifestyle habits according to the recommendations, and reaching a mutual agreement on what to do. Their reasoning revealed that they recognise that genuine change can best be achieved at the micro-system level, where the patient and professional meet as equals.

The novelty of this study lies in using theory and the engagement of end-users’ pre-implementation to identify barriers and facilitating factors and link them to the implementation strategy [23]. Taking part in this qualitative study allowed HCPs to reflect and inter-professionally discuss a healthy lifestyle-promoting practice, which activates a change process. After conducting the interviews and FGDs, data from field notes and transcripts were directly forwarded to the external facilitators and an advisory board (managers at the highest management levels in the PHC regions) for selecting and adapting the implementation activities according to the barriers and facilitating factors identified by the HCPs [25]. Several identified barriers (e.g., lack of knowledge of clinical guidelines, uncertainty in practicalities, and challenges in addressing and achieving behaviour change) are similar to those previously described [14, 16]. A recent qualitative study underscored the importance of HCPs in understanding the need for organisational change and how it benefits patients [24]. In our study the HCPs expressed a strong desire for a healthy lifestyle-promoting practice, meeting the needs of their patients and their commitment to work with health promotion. These expectations may contribute to the inner motivation of HCPs to participate in changing health-promoting practices. This study may provide general knowledge on engaging target groups and understanding their perspectives to select and tailor an implementation strategy according to contextual conditions, the needs of recipients, and the clinical intervention, as suggested by the i-PARIHS framework [50]. Future research is warranted to study the role of facilitators and the specific mechanisms contributing to the successful implementation of primary care intervention.

### Study strengths and limitations

The strengths and limitations of this study are discussed in terms of the model of trustworthiness [41, 51]. Credibility was strengthened by the carefully designed research process and detailed description of how each phase should be performed. All authors are experienced qualitative researchers with different perspectives based on sex, age, research fields, and professional background (two with clinical experience from PHC as physiotherapists). The information power model guided the sample size of the study participants [40, 43]. Informational power was deemed sufficient based on the specific and purposeful sample, the use of a theoretical framework, and the quality of the dialogue. Although we sought a variation of HCPs in the FGDs, a study limitation is that no physician took part in two of five FGDs, which was perceived as disappointing but did not surprise the group members. While no reasons were given, the lack of time to participate in research is a well-known and critical challenge in recruiting HCPs [52, 53]. We used a highly structured approach to strengthen credibility, including pre-testing interview guides and an analysis matrix, recording and transcribing interviews and FGDs, and a detailed description of the analytic process. We employed open-ended questions and interview guides and included areas from all CFIR domains; however, there may be other relevant unknown barriers and facilitating factors.

Most individual interviews were held by phone or digitally, which may be a limitation. Telephone interviews have generally been considered inferior to face-to-face interviews because of the inability to respond to visual cues that may hamper data quality [54]. This assumption, however, has been questioned, and a growing number of studies have emphasised the convenience and methodological strengths of conducting qualitative interviews by phone [55, 56]. The comprehensive data enhanced dependability gathered through interviews and FGDs, suggesting that the data collection process was successful. Because of the rigorous and rich data, we plan to present the findings in two scientific articles: one concerning HCPs’ expectations for implementing a healthy lifestyle-promotion practice (the present article) and another focusing on the HCPs’ expectations of support. To achieve high dependability we included tables and attachments that explain the categorisation process. Dependability was strengthened by the theoretical starting point, structured deductive analytic method, independent coding, and continuing dialogue among the co-researchers. The selection of the determinant framework (i.e. the CFIR) was deemed appropriate for the study aim, namely, exploring facilitating factors and barriers to implementing a healthy lifestyle-promoting practice. In addition, the present framework facilitated the development of interview guides and data analysis. This use of theory in empirical research has been suggested to ensure a more integrated body of knowledge on effective implementation [31]. An inductive analysis followed the deductive data analysis to describe the content of CFIR constructs at a deeper level (i.e., what and how the HCPs expressed their expectations and perceptions of the implementation). The dialogue among the co-researchers was mainly concerned with coding difficulties, according to the CFIR.

When reporting the findings, codes related to the construct Relative advantage are presented along with those in the Tension for change construct. These constructs were perceived to reflect similar features when analysing the comments of the HCPs. The CFIR acknowledges the importance of patient characteristics in implementation but lists the construct of Patient needs and resources in the Outer setting [22]. Listing the patient in the Outer setting may conflict with the past decades' shift to person-centred care in which the values and preferences of each individual are the starting point for all further aspects of care [57]. In this study the HCPs underlined the centrality of patients and that the implementation meets the needs of the targeted groups. Confidence about the trustworthiness of this research is based on the presentation of the coding matrix, the tables specifying categories, and a detailed description of the analytic process. To ensure confirmability and authenticity each sub-category has one citation, and citations from different participants are used [44]. These citations demonstrate the link between results and data, implying adequate authenticity. No member check was performed, but preliminary results were presented at a meeting of PHC managers. By providing information on sampling strategy, context, and participant characteristics, readers can assess whether the results drawn from this sample are transferrable to other similar PHC contexts. The informants were recruited from five PHC centres in the Act in Time project. These centres vary in size and urban/rural and patient demographics, implying that the findings may generalise to other primary care settings in Western countries.

## Conclusions

Implementing a healthy lifestyle-promoting practice in a PHC setting was valued by HCPs, although changing routine practice was challenging. Adopting a health-promotion practice using individually targeted lifestyle interventions needs to be driven by the inner motivation of the HCPs and grounded in a collective understanding of the purpose of changing practice, outlining obvious benefits for their patients. The findings on the identified barriers and facilitating factors will be used to select and adapt the implementation activities to a specific context (i.e., the PHC intervention centres) in the ongoing Act in Time project.

## Supplementary Information

**Additional file 1. **Interview guides.

## Data Availability

The datasets generated and analysed during the current study are not publicly available because they contain information that could compromise the integrity of the participants but are available from the corresponding author at reasonable request.

## Abbreviations

CFIR: Consolidated Framework for Implementation Research
FGDs: Focus group discussions
HCPs: Health care professionals
PHC: Primary health care

## References

[1] Lim SS, Vos T, Flaxman AD, Danaei G, Shibuya K, Adair-Rohani H, Amann M, Anderson HR, Andrews KG, Aryee M (2012). A comparative risk assessment of burden of disease and injury attributable to 67 risk factors and risk factor clusters in 21 regions, 1990–2010: a systematic analysis for the Global Burden of Disease Study 2010. Lancet (London, England).

[2] OECD (2020). Health at a Glance: Europe 2020: State of Health in the EU Cycle.

[3] Beaglehole R, Bonita R, Horton R, Adams C, Alleyne G, Asaria P, Baugh V, Bekedam H, Billo N, Casswell S (2011). Priority actions for the non-communicable disease crisis. Lancet (London, England).

[4] Yusuf S, Joseph P, Rangarajan S, Islam S, Mente A, Hystad P, Brauer M, Kutty VR, Gupta R, Wielgosz A (2020). Modifiable risk factors, cardiovascular disease, and mortality in 155 722 individuals from 21 high-income, middle-income, and low-income countries (PURE): a prospective cohort study. Lancet (London, England).

[5] Marques-Vidal P (2020). Comparison of lifestyle changes and pharmacological treatment on cardiovascular risk factors. Heart.

[6] Álvarez-Bueno C, Cavero-Redondo I, Martínez-Andrés M, Arias-Palencia N, Ramos-Blanes R, Salcedo-Aguilar F (2015). Effectiveness of multifactorial interventions in primary health care settings for primary prevention of cardiovascular disease: a systematic review of systematic reviews. Prev Med.

[7] Journath G, Hammar N, Vikström M, Linnersjö A, Walldius G, Krakau I, Lindgren P, de Faire U, Hellenius ML (2020). A Swedish primary healthcare prevention programme focusing on promotion of physical activity and a healthy lifestyle reduced cardiovascular events and mortality: 22-year follow-up of 5761 study participants and a reference group. Br J Sports Med.

[8] Sisti LG, Dajko M, Campanella P, Shkurti E, Ricciardi W, de Waure C (2018). The effect of multifactorial lifestyle interventions on cardiovascular risk factors: a systematic review and meta-analysis of trials conducted in the general population and high risk groups. Prev Med.

[9] Arnett DK, Khera A, Blumenthal RS (2019). 2019 ACC/AHA Guideline on the Primary Prevention of Cardiovascular Disease: Part 1, Lifestyle and Behavioral Factors. JAMA Cardiol.

[10] Cosentino F, Grant PJ, Aboyans V, Bailey CJ, Ceriello A, Delgado V, Federici M, Filippatos G, Grobbee DE, Hansen TB (2020). 2019 ESC Guidelines on diabetes, pre-diabetes, and cardiovascular diseases developed in collaboration with the EASD. Eur Heart J.

[11] Nationella riktlinjer för prevention och behandling vid ohälsosamma levnadsvanor. Stöd för styrning och ledning (In Swedish). https://www.socialstyrelsen.se/globalassets/sharepoint-dokument/artikelkatalog/nationella-riktlinjer/2018-6-24.pdf. Accessed 21 Sept 2022.

[12] Kardakis T, Jerden L, Nystrom ME, Weinehall L, Johansson H (2018). Implementation of clinical practice guidelines on lifestyle interventions in Swedish primary healthcare - a two-year follow up. BMC Health Serv Res.

[13] McElwaine KM, Freund M, Campbell EM, Bartlem KM, Wye PM, Wiggers JH (2016). Systematic review of interventions to increase the delivery of preventive care by primary care nurses and allied health clinicians. Implement Sci.

[14] Keyworth C, Epton T, Goldthorpe J, Calam R, Armitage CJ (2020). Delivering opportunistic behavior change interventions: a systematic review of systematic reviews. Prev Sci.

[15] Zurynski Y, Smith C, Siette J, NicGiollaEaspaig B, Simons M, Knaggs GT (2021). Identifying enablers and barriers to referral, uptake and completion of lifestyle modification programmes: a rapid literature review. BMJ Open.

[16] Wändell PE, de Waard AM, Holzmann MJ, Gornitzki C, Lionis C, de Wit N, Søndergaard J, Sønderlund AL, Kral N, Seifert B (2018). Barriers and facilitators among health professionals in primary care to prevention of cardiometabolic diseases: a systematic review. Fam Pract.

[17] Rogers HL, Pablo Hernando S, Núñez-Fernández S, Sanchez A, Martos C, Moreno M, Grandes G. Barriers and facilitators in the implementation of an evidence-based health promotion intervention in a primary care setting: a qualitative study. J Health Organ Manag 2021, ahead-of-print(ahead-of-print). 10.1108/JHOM-12-2020-0512 PMC9136863 34464035

[18] Rogers L, De Brun A, McAuliffe E (2020). Defining and assessing context in healthcare implementation studies: a systematic review. BMC Health Serv Res.

[19] Keyworth C, Epton T, Goldthorpe J, Calam R, Armitage CJ (2019). 'It's difficult, I think it's complicated': Health care professionals' barriers and enablers to providing opportunistic behaviour change interventions during routine medical consultations. Br J Health Psychol.

[20] World Health Organization. Primary health care: closing the gap between public health and primary care through integration. https://www.who.int/publications/i/item/primary-health-care-closing-the-gap-between-public-health-and-primary-care-through-integration. Accessed 21 Sept 2022.

[21] Nilsen P, Bernhardsson S (2019). Context matters in implementation science: a scoping review of determinant frameworks that describe contextual determinants for implementation outcomes. BMC Health Serv Res.

[22] Damschroder LJ, Aron DC, Keith RE, Kirsh SR, Alexander JA, Lowery JC (2009). Fostering implementation of health services research findings into practice: a consolidated framework for advancing implementation science. Implement Sci.

[23] Colquhoun HL, Squires JE, Kolehmainen N, Fraser C, Grimshaw JM (2017). Methods for designing interventions to change healthcare professionals' behaviour: a systematic review. Implement Sci.

[24] Nilsen P, Seing I, Ericsson C, Birken SA, Schildmeijer K (2020). Characteristics of successful changes in health care organisations: an interview study with physicians, registered nurses and assistant nurses. BMC Health Serv Res.

[25] Strid EN, Wallin L, Nilsagård Y (2022). Implementation of a Health Promotion Practice Using Individually Targeted Lifestyle Interventions in Primary Health Care: Protocol for the “Act in Time” Mixed Methods Process Evaluation Study. JMIR Res Protoc.

[26] Harvey G, Kitson A (2015). Implementing evidence-based practice in healthcare: a facilitation guide.

[27] Cranley LA, Cummings GG, Profetto-McGrath J, Toth F, Estabrooks CA (2017). Facilitation roles and characteristics associated with research use by healthcare professionals: a scoping review. BMJ Open.

[28] Kotter JP (1996). Leading change.

[29] Odell K (2019). Förändringshandboken: för ledare och medarbetare (In Swedish).

[30] Glasgow RE, Goldstein MG, Ockene JK, Pronk NP (2004). Translating what we have learned into practice - Principles and hypotheses for interventions addressing multiple behaviors in primary care. Am J Prev Med.

[31] Nilsen P (2015). Making sense of implementation theories, models and frameworks. Implement Sci.

[32] Kirk MA, Kelley C, Yankey N, Birken SA, Abadie B, Damschroder L (2016). A systematic review of the use of the Consolidated Framework for Implementation Research. Implement Sci.

[33] Skolarus TA, Lehmann T, Tabak RG, Harris J, Lecy J, Sales AE (2017). Assessing citation networks for dissemination and implementation research frameworks. Implement Sci.

[34] Birken SA, Powell BJ, Shea CM, Haines ER, Kirk MA, Leeman J, Rohweder C, Damschroder L, Presseau J (2017). Criteria for selecting implementation science theories and frameworks: results from an international survey. Implement Sci.

[35] van der Heiden W, Lacroix J, Moll van Charante EP, Beune E (2022). GPs' views on the implementation of combined lifestyle interventions in primary care in the Netherlands: a qualitative study. BMJ Open.

[36] Rogers HL, Fernández SN, Pablo Hernando S, Sanchez A, Martos C, Moreno M, Grandes G (2021). "My Patients Asked Me If I Owned a Fruit Stand in Town or Something." Barriers and Facilitators of Personalised Dietary Advice Implemented in a Primary Care Setting. J Pers Med.

[37] Martinez C, Bacigalupe G, Cortada JM, Grandes G, Sanchez A, Pombo H, Bully P, Grp PVS (2017). The implementation of health promotion in primary and community care: a qualitative analysis of the ` Prescribe Vida Saludable' strategy. BMC Fam Pract.

[38] Moullin JC, Dickson KS, Stadnick NA, Albers B, Nilsen P, Broder-Fingert S, Mukasa B, Aarons GA (2020). Ten recommendations for using implementation frameworks in research and practice. Implement Sci Commun.

[39] Waltz TJ, Powell BJ, Fernandez ME, Abadie B, Damschroder LJ (2019). Choosing implementation strategies to address contextual barriers: diversity in recommendations and future directions. Implement Sci.

[40] Krueger RaC, M. Focus Groups. A Practical Guide for Applied Research. 4th edition. Thousand Oaks: SAGE publication, Inc. 2009.

[41] Denzin NK, Lincoln YS (2000). Handbook of qualitative research.

[42] Elo S, Kyngas H (2008). The qualitative content analysis process. J Adv Nurs.

[43] Malterud K, Siersma VD, Guassora AD (2016). Sample Size in Qualitative Interview Studies: Guided by Information Power. Qual Health Res.

[44] Eldh AC, Arestedt L, Bertero C (2020). Quotations in qualitative studies: reflections on constituents, custom, and purpose. Int J Qual Methods.

[45] World Medical Association (2013). World Medical Association Declaration of Helsinki: ethical principles for medical research involving human subjects. JAMA.

[46] Tong A, Sainsbury P, Craig J (2007). Consolidated criteria for reporting qualitative research (COREQ): a 32-item checklist for interviews and focus groups. Int J Qual Health Care.

[47] Socialdepartementet, (Ministry of social affairs): God och nära vård. En reform för ett hållbart hälso- och sjukvårdssystem. SOU 2020:19. (Good and close care. A reform for a sustainable health care system). (In Swedish). Stockholm: Socialdepartementet, English Ministry of social affairs. 2020.

[48] Lonnberg L, Damberg M, Revenas A (2022). Lifestyle counselling - a long-term commitment based on partnership. BMC Primary Care.

[49] Carmona C, Crutwell J, Burnham M, Polak L, Guideline C (2021). Shared decision-making: summary of NICE guidance. BMJ.

[50] Harvey G, Kitson A (2016). PARIHS revisited: from heuristic to integrated framework for the successful implementation of knowledge into practice. Implement Sci.

[51] Kyngas H, Kaariainen M, Elo S, Kyngäs H, Mikkonen K, Kääriäinen M (2020). The Trustworthiness of Content Analysis. The Application of Content Analysis in Nursing Science Research.

[52] Hysong SJ, Smitham KB, Knox M, Johnson KE, SoRelle R, Haidet P (2013). Recruiting clinical personnel as research participants: a framework for assessing feasibility. Implement Sci.

[53] Flynn R, Albrecht L, Scott SD (2018). Two Approaches to Focus Group Data Collection for Qualitative Health Research: Maximizing Resources and Data Quality. Int J Qual Methods.

[54] Novick G (2008). Is there a bias against telephone interviews in qualitative research?. Res Nurs Health.

[55] Vogl S (2013). Telephone versus face-to-face interviews: Mode effect on semistructured interviews with children. Sociol Methodol.

[56] Cachia M, Millward L (2011). The telephone medium and semi-structured interviews: a complementary fit. Qual Res Organ.

[57] World Health Organization (2015). WHO global strategy on people-centred and integrated health services: interim report.

