# Supplementary material for: Expectations on implementation of a health promotion practice using individually targeted lifestyle interventions in primary health care: a qualitative study
Source: BMC Prim Care. 2023 Jun 16;24:122. doi: 10.1186/s12875-023-02079-5 (PMC10273504; doi:10.1186/s12875-023-02079-5)
Supplement: Supplementary file 1 — Additional file 1. Interview guides. [file 12875_2023_2079_MOESM1_ESM.pdf]

Interview guides

Follow-up questions on perceptions on barriers and facilitating factors

Managers

- Thoughts and expectations on the described/planned health-promotion practice using individually targeted lifestyle interventions
- How do you think your employees value health promotion and their ability to work with the health-promotion practice?
- How do you think the patients will perceive the health-promoting practice?
- How important is it to ask patients on lifestyle habits and provide support to change unhealthy lifestyle habits in the PHC setting?
- How does the health-promotion practice align with healthcare policies, missions and goals?
- How would you describe the engagement of higher manager levels?
- What driving forces would motivate you to lead this work and be a part its success?
- How will the health-promotion practice be handled among other, competing, missions and tasks?
- How can the health-promoting practice be integrated in ongoing practice?
- What changes are needed to implement the practice at the PHC centre?
- Do you have a plan for the implementation at your PHC centre?
- What do you expect your employees to do?
- What do you think your employees may need to start working according to the health-promotion practice?
- How will you engage your employees?
- What kind of support may you need?

Internal facilitators

- Thoughts and expectations on the described/planned health-promotion practice using individually targeted lifestyle interventions, will it make a difference?
- Do you known the clinical guidelines and screening form, and what are your thoughts of using it?
- How important is it that this health-promoting practice will be implemented? (For you, for your patients)
- How does the health-promoting practice serve your patients' needs?
- How well does the health-promoting practice fit with what you already do today?
- How could it be more integrated?
- What changes might be needed so that you and your colleagues can work according to this health-promoting practice?
- How will this health-promoting practice be prioritised among other, competitive, work tasks?
- What may trigger your colleagues to work more health-promoting?
- How will you start?
- Your mission as an internal facilitator is to support your colleagues at the PHC centre, how confident do you feel?
- What kind of support do you think you will have to provide to your colleagues?
- How do you think your manager will value your efforts as internal facilitator?
- What kind of support do you think you will need to accomplish your mission as an internal facilitator?

- What kind of support do you think you will need from the external facilitators?

Focus group discussions with healthcare professionals

- Thoughts and expectations on the described/planned health-promotion practice using individually targeted lifestyle interventions from the perspective of their profession
- How important is it that this health-promoting practice will be a more pronounced part of your work?
- How does this health-promoting practice serve your patients' needs?
- How does this health-promoting practice fit with what you already do today?
- How could the health-promoting practice be more integrated in your daily practice?
- How would you start?
- What changes might be needed so that you and your colleagues can work according to this health-promoting practice?
- How will this health-promoting practice be prioritised among other, competitive, work tasks?
- What would motivate your to work more health promoting according to this practice?
- What would trigger you to ask your patients about their lifestyle habits and health?
- What would facilitate for you to work according to this health-promoting practice?
- What kind of support to do expect from your manager or others to succeed with this?
- How confident do you feel in your ability to implement this practice?
